# Instruments to measure e-cigarette related constructs: a systematic review

**DOI:** 10.1186/s12889-022-13510-4

**Published:** 2022-06-07

**Authors:** Eunhee Park, Misol Kwon, Thomas Chacko, Yanjun Zhou, Chiahui Chen, Maciej L. Goniewicz, Chin-Shang Li, Yu-Ping Chang

**Affiliations:** 1 University at Buffalo, School of Nursing, 3435 Main St. University at Buffalo, Wende Hall, Buffalo, NY 14214-8013 USA; 2 Roswell Park Comprehensive Cancer Center, Elm and Carlton Streets, Buffalo, NY 14263 USA

**Keywords:** Instrument, Electronic cigarettes, Survey, Psychometrics

## Abstract

**Background:**

Electronic cigarettes (e-cigarettes) are relatively new tobacco products that are attracting public attention due to their unique features, especially their many flavor options and their potential as an alternative to cigarettes. However, uncertainties remain regarding the determinants and consequences of e-cigarette use because current research on e-cigarettes is made more difficult due to the lack of psychometrically sound instruments that measure e-cigarette related constructs. This systematic review therefore seeks to identify the instruments in the field that are designed to assess various aspects of e-cigarette use or its related constructs and analyze the evidence presented regarding the psychometric properties of the identified instruments.

**Methods:**

This systematic review utilized six search engines: PubMed, Medline, CINAHL, PsycINFO, Web of Science, and EMBASE, to identify articles published in the peer-reviewed journals from inception to February 2022 that contained development or validation processes for these instruments.

**Results:**

Eighteen articles describing the development or validation of 22 unique instruments were identified. Beliefs, perceptions, motives, e-cigarette use, and dependence, were the most commonly assessed e-cigarette related constructs. The included studies reported either construct or criterion validity, with 14 studies reporting both. Most studies did not report the content validity; for reliability, most reported internal consistencies using Cronbach’s alpha, with 15 instruments reporting Cronbach’s alpha > 0.70 for the scale or its subscales.

**Conclusions:**

Twenty-two instruments with a reported development or validation process to measure e-cigarette related constructs are currently available for practitioners and researchers.

This review provides a guide for practitioners and researchers seeking to identify the most appropriate existing instruments on e-cigarette use based on the constructs examined, target population, psychometric properties, and instrument length. The gaps identified in the existing e-cigarette related instruments indicate that future studies should seek to extend the validity of the instruments for diverse populations, including adolescents. Instruments that explore additional aspects of e-cigarette use and e-cigarette related constructs to help build a strong theoretical background and expand our current understanding of e-cigarette use and its related constructs, should also be developed.

## Background

Electronic cigarette (e-cigarette) use is emerging as a major item on the public health agenda, attracting both greater attention from researchers, and intense scrutiny from the popular media. A significant increase in the prevalence of e-cigarette use among US adults has been reported since 2010 [1–3]; in 2019, 4.5% of adults in the US self-reported using e-cigarettes, of whom 36.9% identified as dual users [4] Among youth, steep rises in nicotine vaping and e-cigarette product use have resulted in an overall increase in the use of tobacco products. In 2020, 19.5% of high school students and 4.7% of middle school students used electronic nicotine delivery systems (ENDS) [4, 5].

Developed to closely approximate the sensory experience of smoking combustible cigarettes, e-cigarettes produce an aerosol by heating a liquid containing a solvent (generally vegetable glycerin, propylene glycol, or a mixture of the two), one or more flavorings, and nicotine, although liquids containing no nicotine are available on the market for some devices [6]. E-cigarettes have gained considerable popularity among both youth and adults in recent years in spite of the dearth of research into the devices’ safety, effects, and efficacy [7]. Hence, while the research regarding the potential health effects of e-cigarette use is still in its infancy, researchers are beginning to try to understand people’s perceptions, reasons, and behaviors in order to better understand their use of e-cigarettes.

Despite reports that e-cigarettes emit substantially lower levels of carcinogens and thus represent a safer alternative to combustible cigarettes [8], young people who use e-cigarettes have shown increased risk of trying combustible cigarettes [9]. Moreover, with hundreds of e-cigarette brands already on the market, vaping products are evolving rapidly in terms of their mechanisms, engineering, design, and usability, all of which are aimed at boosting their appeal for curious youngsters and thus posing an additional concern as sales of these products continue to rise. This raised serious concerns for young people’ health because young e-cigarette users are reported to have more physical and mental health issues [10]. E-cigarettes contain nicotine, and exposure to toxicants, such as nicotine, has deleterious effects on the developing brain [11, 12]. Furthermore, there are substances, such as formaldehyde, and acetaldehyde, which cause cancers [13, 14].

As research in this area increases, it is vital that studies that focus on e-cigarette use are able to utilize reliable and valid e-cigarette use measures when assessing their results. Major gaps remain in our knowledge of the effects and potential hazards posed by e-cigarettes that require extensive research, particularly when it comes to exploring major factors associated with e-cigarette use such as the motivators influencing the decision to use e-cigarettes and the consequences of e-cigarette use. However, there are some unique challenges for those developing new instruments to measure these constructs. E-cigarettes are relatively new and rapidly evolving products and thus, there is significant variability in the products currently on the market, including refillable options as well as pens, pods, and other configurations; the different patterns of e-cigarette use include experimentation, regular use, and dual use. However, presently, there is limited information regarding the validity of the various instruments developed to examine the multi-faceted issues involved and a clear need to evaluate the measurement properties of each of these instruments.

To date, there have been no systematic evaluations of the available evidence supporting the measurement properties of these validated instruments for e-cigarette use. Hence, the purpose of this systematic review is to review and synthesize validated survey instruments in the literature that are specifically designed to explore e-cigarette related constructs. In this context, *survey instrument* refers to the data collection tool that measures a construct in survey methods [15]. In addition, *e-cigarette-related construct* is defined as a construct chosen by researchers to explore and analyze the mechanisms of e-cigarette use or the phenomena associated with its use in survey studies. Construct is defined as “an image, idea, or theory, especially a complex one formed from a number of simpler elements ([16] , p.1),” and it usually refers to the latent construct that is inferred by observing indicating behaviors. For example, constructs such as motivation, dependence, perceived harms and benefits, and dependency of e-cigarette use are often explored in e-cigarette survey research, thus, these are typical examples that we would expect to include as e-cigarette-related constructs. Specifically, this study aims to provide an overview of existing instruments developed for measuring e-cigarette-related constructs including the development or validation process and psychometric properties, thus bridging a serious gap in e-cigarette survey research. Our ultimate goal is to assist both researchers in the field and clinicians to make informed choices when selecting an appropriate instrument for the measurement of e-cigarette use.

## Methods

This systematic review was registered with the PROSPERO international prospective register of systematic reviews and was conducted following the guidelines laid out in the Preferred Reporting Items for Systematic Reviews and Meta-Analyses (PRISMA) [17].

### Search strategy

A systematic search was conducted using six electronic databases: PubMed, Medline, Cumulative Index of Nursing and Allied Health Literature (CINAHL), PsycINFO, Web of Science, and Excerpta Medica dataBASE (EMBASE) from inception to February 2022. In addition, the works cited in the reviews and the references in the retrieved articles were screened. To broaden our search results, we entered our search terms using two categories, namely *e-cigarette* and *instrument*. The following search keywords or Medical Subject Headings (MeSH) were thus used: *vaping device* or *vape* or *electronic cigarettes* or *e-cigarettes* or *e-liquid* or *electronic nicotine delivery systems* [mesh] AND *psychometrics* [mesh] or *questionnaires* or *surveys* or *surveys and questionnaires* [mesh]. It is important to note that we did not specify search terms that would limit the constructs related to e-cigarette use. For example, we did not utilize terms such as *motivation*, *belief*, *symptom*, *perceived harms* or *benefits*, *consequences*, and *behavior* even though we were aware of instruments that measured some of these constructs as they are often used in e-cigarette survey research. By adopting this approach, we were able to explore the extent of the constructs that are assessed by validated instruments.

A filter was applied to retrieve articles published in English, but no language restriction was applied for the instruments. In addition, a snowballing technique was used to suggest additional searches: if the article referred to earlier articles that described the process of development or validation of the instruments, we also retrieved those articles and checked their eligibility for inclusion in this review.

### Inclusion and exclusion criteria

Predetermined inclusion criteria were applied to select relevant studies, which included (1) Articles reporting the development and/or validation process for survey instruments designed to measure electronic cigarette related constructs (e.g., motivation, dependence, perceived harms and benefits, consequences, and behavior); (2) Full text articles published in peer-reviewed research journals; and (3) Articles published in English, where the instruments were translated into English for the purpose of the analysis. Likewise, the study specified the following exclusion criteria: (1) studies that are not empirical; (2) single-item instruments; (3) the validation or development process of survey instruments were not reported; and (4) instruments designed for use in laboratory settings.

### Selection process

Applying the aforementioned inclusion and exclusion criteria, two authors screened relevant titles and abstracts independently. Studies that met the criteria were accessed and independently reviewed multiple times by all the authors and discrepancies were reconciled through consensus discussions.

### Data extraction

To provide an overview of the instruments and the psychometric properties of each, the following coding schemes were used: (1) Basic information on the instruments, including the name of the instrument, the name of the first author, the constructs that the instrument is designed to assess, the country where the study took place, the theoretical background of the instrument, the mode of administration, the completion time, and the response options (Table 1); and (2) The psychometric properties of the instruments, including the constructs, sub-constructs, various types of reliability reported (e.g., internal consistency, test-retest reliability) and validity (construct, content, and criterion validity) tested (Table 2).

**Table 1:** Overview of Instruments

Broad categories of constructs	Constructs	Theory	Instruments	Reference articles	Target age	Reliability (Cronbach’s alpha > 0.70)	Validity			# of items
							Content	Construct	Criterion	
Beliefs/ perception/attitudes	Outcome expectancies	Motivation theories	Revised youth e-cigarette outcome expectancies	Pokhrel et al., 2018 [18]	18–25	O	–	O	O	43
		Motivation theories	Revised youth EC outcome expectancies (short)	Pokhrel et al., 2018 [18]	18–25	O	–	O	O	12
		–	Adolescent E-Cigarette Consequences Questionnaire (AECQ)	Cristello et al., 2020 [19]	High school students (Mean = 14.90)	–	–	O	–	18
	Vaping expectancies, sensory expectancies	–	Sensory E-cigarette Expectancies Scale (SEES)	Morean et al., 2019 [20]	≥18	O	–	O	O	9
		Social learning theory	Short Form Vaping Consequences Questionnaire (S-VCQ)	Morean & L’Insalata, 2017 [21]	≥18	O	–	O	O	21
	Perceived risk and benefits of e-cigarettes	–	Perceived Risk and Benefits of E-cigarette use (RABE)	Copeland et al., 2017 [22]	≥18	O	–	O	–	30
		–	Conditional Risk Assessment of Electronic Cigarette Perceptions	Chaffee et al., 2015 [23]	13–18	–	–	O	O	19
	Comparative beliefs of e-cig use and cigarette smoking	Theory of planned behavior (TPB)	Comparing E-Cigarette and Cigarettes Questionnaire (CEAC)	Hershberger et al., 2017 [24]; Kale et al., 2020 [25]	≥18	O	–	O	O	10
	E-cigarette expectancies compared to cigarette smoking	–	E-cigarette-specific Brief Smoking Consequences Questionnaire-Adult (BSCQ-A)	Hendricks et al., 2015 [26]	≥19	O (>.67)	–	O	O	25
	Perceived harms compared with cigarettes	–	Direct and indirect measures of perceived harm of e-cigarettes and smokeless tobacco compared with smokeless tobacco	Persoskie et al., 2017 [27]	12–17	–	–	–	O	2
	Perceived harms and social norms in the use of e-cigarettes and smokeless tobacco	TPB & integrated model of behavior change	Perceived harms and social norms in the use of electronic cigarettes	Waters et al., 2017 [28]	≥18	O	–	O	O	15
	Expectancies of combined e-cigarette and alcohol use	–	Nicotine and Other Substance Interaction Expectancy Questionnaire E-cig Revised version (NOSIE-ER)	Hershberger et al., 2016 [29]	≥21	O	–	O	O	8
	Attitudes toward e-cig use	–	Electronic cigarette attitudes survey (ECAS)	Diez et al., 2019 [30]	14–19	O	–	O	–	12
Motives	Motivations for e-cigarette experimentation	–	Motivations for e-cigarette experimentation**	Penzes et al., 2016 [31]	≥18 (non-users; young adults)	O (>.68)	–	O	O	27
Use	Susceptibility to future use	–	Susceptibility scale	Cole et al., 2019 [32]	14–17	–	–	–	O	3
		–	Susceptibility to four product classes (e-cigarettes, cigars, hookah and cigarettes)	Carey et al., 2018 [33]	10–18	O	–	O	O	3
	Habitual e-cigarette use	–	Self-report Habit Index (SRHI)	Morean et al., 2018 [34]	≥18	O	–	O	O	12
Symptoms	E-cigarette craving	–	Questionnaire of Vaping Craving (QVC)	Dowd et al., 2019 [35]	≥18	O	–	O	–	10
	E-cigarette dependence	–	Penn State Electronic Cigarette Dependence Index (PS-ECDI).	Piper et al., 2019 [36]; Foulds et al., 2015 [37]	≥18	O	O	O	O	10
		–	E-cigarette Fagerström Test of Cigarette Dependence (e-FTCD)	Piper et al., 2019 [36]	≥18	–	–	O	–	6
		–	E-cigarette Wisconsin Inventory of Smoking Dependence Motives (e-WISDM)	Piper et al., 2019 [36]	≥18	O	–	O	O	37
		–	Fagerström Test for Nicotine Dependence applied to Vaping (FTND-V)	Browne & Todd, 2018 [38]	≥17	–	–	O	–	9

*Abbreviations*: *TPB* Theory of planned behavior

O = indicates the studies addressed reported reliability or validity

** = freshman/sophmore was reported

**Table 2:** Basic Information about the Instruments

Instrument	Author & year	Country	Age range	Theory	Mode of administration	Completion time	Final number of items	Response options
Adolescent E-Cigarette Consequences Questionnaire (AECQ)	Cristello et al., 2020 [19]	USA	High school students (Mean = 14.90)	NR	In person	NR^a^	18	1–5 Likert
Electronic cigarette attitudes survey (ECAS)	Diez et al., 2019 [30]	USA	14–19	NR	In-person	NR^a^	12	1–5 Likert
Sensory E-cigarette Expectancies Scale (SEES)	Morean et al., 2019 [20]	USA	≥18	NR	Online	NR^a^	9	1–5 Likert
E-cigarette Fagerström Test of Cigarette Dependence (e-FTCD)	Piper et al., 2019 [36]	USA	≥18	NR	NR	NR	6	NR
E-cigarette Wisconsin Inventory of Smoking Dependence Motives (e-WISDM)	Piper et al., 2019 [36]	USA	≥18	NR	NR	NR	37	NR
Penn State Electronic Cigarette Dependence Index (PS-ECDI).	Piper et al., 2019 [36]; Foulds et al., 2015 [37]	USA	≥18	NR	Online	NR	10	Mixed
Questionnaire of Vaping Craving (QVC)	Dowd et al., 2019 [35]	USA	≥18	NR	Online	> 10 min.	10	1–7 Likert
Susceptibility to future use	Cole et al., 2019 [32]	Canada	14–17^b^	NR	NR	NR	3	NR
Revised youth EC outcome expectancies	Pokhrel et al., 2018 [18]	USA	18–25	Motivation Theories	NR	NR	43	1–10 Likert
Revised youth EC outcome expectancies (Short version)	Pokhrel et al., 2018 [18]	USA	18–25	Motivation Theories	NR	NR	12	1–10 Likert
Self-report habit index (SRHI e-cigarette)	Morean et al., 2018 [34]	USA	≥18	NR	Online	NR	12	1–2 Likert
Susceptibility to four product classes (EC, cigars, hookah and cigarettes)	Carey et al., 2018 [33]	USA	10–18	NR	Online	NR^a^	3	1–4 Likert
Fagerström Test for Nicotine Dependence applied to Vaping (FTND-V)	Browne & Todd, 2018 [38]	Australia	≥17	NR	Online	NR	9	Mixed
Comparing EC and Cigarettes Questionnaire (CEAC)	Hershberger et al., 2017 [24]; Kale et al., 2020 [25]	USA; England	≥18	Theory of Planned Behavior	Online	NR	10	1–5 Likert
Perceived harms and social norms in the use of e-cigarettes and smokeless tobacco	Waters et al., 2017 [28]	USA	≥18	Theory of planned behavior & integrated model of behavior change	Online	NR	15	1–9 Likert
Perceived harm of EC and smokeless tobacco with cigarettes	Persoskie et al., 2017 [27]	USA	12–17	NR	NR	NR	2	1–4 Likert
Risk and Benefits of E-cigarettes (RABE)	Copeland et al., 2017 [22]	USA	≥18	NR	Online	NR	30	1–7 Likert
Short Form Vaping Consequences Questionnaire (S-VCQ)	Morean & L’Insalata, 2017 [21]	USA	≥18	Social learning theory	Online	NR	21	NR
Motivations of intention to try EC among non-EC users	Penzes et al., 2016 [31]	Hungary	≥18	NR	Online	NR	27	1–4 Likert
Nicotine and Other Substance Interaction Expectancies-E-cig Revised version (NOSIE-ER)	Hershberger et al.,2016 [29]	USA	≥21	NR	Online	NR	8	True/False
e-cigarette-specific Brief Smoking Consequences Questionnaire (e-cigarette-specific BSCQ-A)	Hendricks et al., 2015 [26]	USA	≥19	Expectancy theory	Online	NR	25	0–9 Likert
Conditional Risk Assessment	Chaffee et al., 2015 [23]	USA	13–18	Social learning theory & health belief model	Online	NR	19	NR

*Abbreviation*: *NR* Not reported, *USA* United States of America

^a^Total study time are reported but instrument-specific time is not reported

^b^Grade 9–12 is reported

### Quality appraisal and risk of Bias

We assessed quality appraisal and risk of bias in the included studies using the COSMIN Risk of Bias checklist (Table 4) [39]. The COSMIN Risk of Bias checklist addresses ten specific domains: (1) PROM design; (2) content validity; (3) structural validity; (4) criterion validity; (5) internal consistency (6) cross-cultural validity/ measurement error; (7) reliability, which is tested with test and retest; (8) measurement error; (9) criterion validity; (10) hypotheses testing for construct validity; and (11) responsiveness. Three review authors (M.K., Y.Z., and C.C.) independently applied the tool to the included studies (*n* = 23) and recorded judgements of risk of bias for each domain (very good, adequate, doubtful, or inadequate). The judgements of “very good” or “adequate” indicated the quality of studies. Following guidance given for COSMIN, we derived an overall summary in the “Quality of appraisal and risk of bias” table for each specific domain, whereby the overall COSMIN for each study was determined by the quality level and risk of bias in ten domains.

## Results

### Search results

**Fig. 1:**
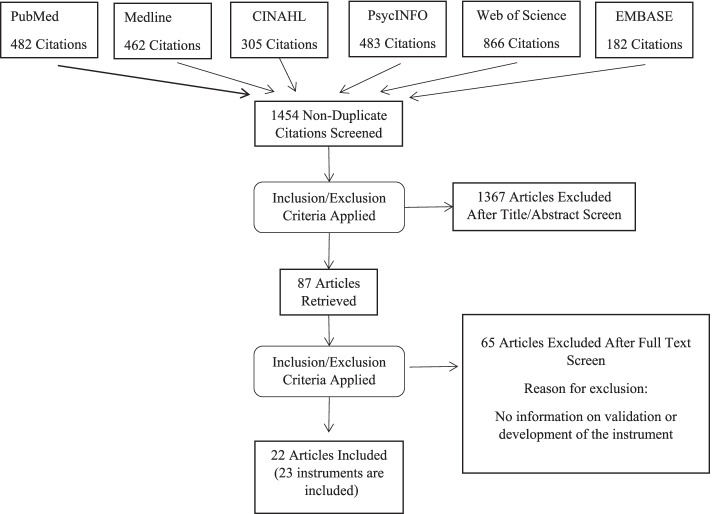
PRIMA Diagram

In total, the search yielded 1454 articles. After two researchers had independently reviewed the titles and abstracts of all articles, 87 were selected to undergo a full text examination, after which the same two researchers independently reviewed their full texts. Of the 87 articles, 65 were excluded as they did not report specific information about the development or validation process utilized. After the full text review based on the eligibility criteria, 23 studies were found to be suitable for inclusion in the current study (see Fig. 1. PRISMA flow chart). One study tested the validity of three instruments that measure the same construct, namely e-cigarette dependence [40]; one study reported the validity of both the long and short versions of the instrument, and revised the youth e-cigarette outcome expectancies respectively [18], and two studies conducted a validity test on the same instrument [18, 37, 40]. Thus, a total of 22 instruments from 23 different studies were analyzed for the current study.

### Overview of instruments

Tables 1 and 2 provide an overview of the characteristics of the instruments presented in the included articles. This section presents an overview of the settings of these studies, the age ranges of their participants, the theoretical frameworks utilized, the modes of administration and durations of the tests, the number of test items, and the response options. This information will guide the users to choose the appropriate instruments depending on their purpose. For example, users will know which instrument exists to measure a construct of their interests, and the target population and the settings under which instruments were validated. Then, the modes of administration, duration of the tests, number of items, and response options can provide additional practical information in choosing which instrument they want to choose.

#### Country and language

Of the 22 instruments, 77.3% (17/22) studies for validation or reliability test in the US; the others were conducted in Canada, Australia, and Hungary [24, 32, 38, 41]. All but one instrument was developed in English. The exception was an instrument originally developed in Hungarian and later translated into English [41]. Although the items of this instrument are available in English, the instrument has not been validated using a cross-cultural translational process.

#### Participants

Regarding the ages of the participants, 72.7% (16/22) of the instruments were designed for use with participants aged 18 years or above; the remaining 27.3% (6/22) were for younger participants who were under 18 years of age [19, 23, 27, 32, 33]. Among the instruments validated for participants aged 18 or above, six instruments were specifically targeted at young adults (18 to 25 years old or college students) [18]. One instrument was validated based on its use with hospitalized patients [26].

#### Administration, number of items, and responses

There were some variability and ambiguity with regard to the modes of administration of the instruments. Although the majority of the tests were administered “online” (63.6%, 14/22), 27.3% (6/22) of the studies did not report the mode of administration [18, 19, 27, 32, 40]. The vast majority of the studies (77.3%, 17/22) did not report the completion time for their instruments; the remaining 13.6% (3/22) specified either the completion time of the instrument (*n* = 1) or the completion time of the study (*n* = 4) [35]. However, the number of items in each instrument can provide a rough estimate of the completion time required. There was a considerable variability with respect to the number of items in the instruments, which ranged from 2 to 55 with a mean of 15.81. Finally, the majority of the instruments (68.2%,15/22) utilized Likert-type response options, varying from 1 to 2 to 1–10, a further 18.2% (4/22) did not report the response options [21, 23, 40], 9.19% (2/22) had mixed response options [37, 38], and 4.5% (1/22) had True/False response options [29].

#### Theoretical background

The majority of the studies (68.2%,15/22) did not describe the theoretical background of their instrumentation clearly (Table 2). The other 22.7% (5/22) did present the theoretical framework underpinning their instruments by including a discussion of the relevant motivation theories, theories of planned behaviors, social learning theory, and/or expectancy theory [18, 21, 23, 24, 26, 28].

#### Constructs

The 22 instruments identified a total of 15 different constructs reflecting the multiple constructs explored in e-cigarette survey research (Table 1). General beliefs and perceptions were identified as the most commonly explored construct, with individual studies specifically including outcome expectancies [18], sensory vaping expectancies [20], and the risks and benefits of e-cigarettes [22, 23].

Fourteen instruments sought to assess beliefs, perceptions, and attitudes about e-cigarettes, specifically comparing the beliefs or perceptions to the beliefs or perceptions about cigarette smoking using constructs such as *comparative beliefs of e-cigarette use* [24], *e-cigarette expectancies compared to cigarette smoking* [29], and *perceived harms compared with cigarettes* [27]. One instrument was designed to assess the *perceived harms and social norms* of both e-cigarette and smokeless tobacco in a single instrument [28], while another was specifically developed to assess the expectancies of simultaneous e-cigarette and alcohol use [29].

*Motivation for e-cigarette experimentation* and *susceptibility to future use* were identified as constructs that were explored by four instruments [41]. These instruments assess motivation or likelihood of using e-cigarettes specifically among non-cigarettes users [32, 33, 41]. Among these instruments, one assessed susceptibility to four different tobacco products, namely e-cigarettes, cigarettes, cigars, and hookahs [33]. Looking at e-cigarette use exclusively, one instrument assessed *habitual e-cigarette use* [34], but no studies that specifically reported the development or validation of instruments assessing e-cigarette use were identified.

The next most commonly assessed constructs were *e-cigarette craving* and *e-cigarette dependence*. One instrument assessed e-cigarette craving based on three sub-constructs, namely desire, intention, and positive outcome [35]. For smoking dependence, four instruments were identified [37, 38, 40]. Of these, three instruments had a one single construct, but one instrument (e-WISDM) has 37 items consisting of 11 sub-constructs: affiliative attachment, affective enhancement, automaticity, loss of control, cognitive enhancement, craving, cue exposure, social/environmental goals, taste, tolerance, and weight control [40].

In terms of the number of sub-constructs within each instrument, most had several sub-constructs (range = 1 to 11; Mean = 3.29). Interestingly, six instruments had no sub-constructs and only a single domain, either *e-cigarette dependence* [38], *susceptibility to future e-cigarette use* [32, 37, 40], or *habitual e-cigarette use* [34].

### Psychometric properties of instruments

In the current study, we examined the psychometric properties of the various instruments included in the 22 relevant instruments identified (Tables 1 & 3).

**Table 3:** Psychometric Properties of the Included Instruments

Author & year	Name of instrument	Construct	Number of sub constructs/sub domains	Reliability		Validity		
				Internal consistency (subscales)	Test-retest reliability	Construct validity	Content validity	Criterion validity
Cristello et al., 2020 [19]	Adolescent E-Cigarette Consequences Questionnaire (AECQ)	Vaping expectancies, sensory expectancies	7 (negative affect reduction, taste and sensorimotor manipulation, social facilitation, weight control, negative physical feelings, boredom reduction, negative social impression)	NR	NR	Test Dimensionality (CFA); Convergent and discriminant validity	NR	NR
Diez et al., 2019 [30]	Electronic Cigarette Attitudes Survey (ECAS)	Attitudes toward e-cig use	1 (attitudes)	0.93	NR	Test Dimensionality (EFA & CFA); Test differences by e-cigarette use status;	NR	NR
Morean et al., 2019 [20]	Sensory E-cigarette Expectancies Scale (SEES)	Sensory vaping expectancies	3 (taste/smell, pleasure/satisfaction, vapor cloud Production)	Subscales: .85–.90	NR	Test Dimensionality (EFA & CFA); Measurement invariance; Convergence with dependence	NR	Concurrent validity (associated with vaping frequency and habitual e-cigarette use)
Piper et al., 2019 [36]	E-cigarette Fagerström Test of Cigarette Dependence (e-FTCD)	E-cigarette dependence measure	1 (dependence)	.51	NR	Test Dimensionality (CFA); Correlation with e-cigarette use measures and; e-cigarette dependence measures	NR	Predictive validity (associated with e-cigarette use)
Piper et al., 2019 [36]	E-cigarette Wisconsin Inventory of Smoking Dependence Motives (e-WISDM)	E-cigarette dependence measure	11 (affiliative attachment, affective enhancement, automaticity, loss of control, cognitive enhancement, craving, cue exposure, social/environmental goals, taste, tolerance, weight control)	.80 (Subscales > 0.80)	NR	Test Dimensionality (CFA); Correlation with e-cigarette use measures and; e-cigarette dependence measures	NR	Predictive validity (associated with e-cigarette use)
Piper et al., 2019 [36]; Foulds et al., 2015 [37]	Penn State Electronic Cigarette Dependence Index (PS-ECDI).	E-cigarette dependence measure	1 (dependence)	.74	NR	Test Dimensionality (CFA); Correlation with e-cigarette use measures and; e-cigarette dependence measures; Test differences by nicotine concentration of products used	Participant Interviews	Predictive validity (associated with e-cigarette use)
Dowd et al., 2019 [35]	Questionnaire of Vaping Craving (QVC)	Craving for e-cigarette measure	3 (desire, intention, positive outcome)	.96	NR	Test Dimensionality (PFA & CFA); Correlation with e-cigarette use, and cigarette use measures; Convergent and discriminant validity	NR	NR
Cole et al., 2019 [32]	Susceptibility scale	Susceptibility to future tobacco products or e-cigarettes use	1 (susceptibility)	NR	NR	NR	NR	Predictive validity (associated with e-cigarette use)
Pokhrel et al., 2018 [18]	Revised youth e-cigarette outcome expectancies	E-cigarette use outcome expectancies	8 (social enhancement, affect regulation, positive sensory experience, negative health consequences, addiction concern, negative sensory experience, positive “smoking” experience, negative social experience)	Subscales: .77–.94	NR	Test Dimensionality (EFA & CFA); Convergence and divergence validity	NR	Concurrent validity (associated with e-cigarette use susceptibility, current e-cigarette use, e-cigarette use dependence)
Pokhrel et al., 2018 [18]	Revised youth EC outcome expectancies (short)	Outcome expectancies	2 (positive outcome expectancies, negative outcome expectancies)	.87–.92	NR	Test Dimensionality (EFA & CFA)	NR	Concurrent validity (associated with e-cigarette use susceptibility, current e-cigarette use, e-cigarette use dependence); construct validity (associated with e-cigarette outcome expectancy factors)
Morean et al., 2018 [34]	Self-report habit index (SRHI)	Habitual e-cigarette use	1 (habitual use)	0.91	NR	Test Dimensionality (CFA & EFA); Measurement invariance	NR	Concurrent validity (associated with increased frequency of e-cigarette use)
Carey et al., 2018 [33]	Susceptibility to four product classes (EC, cigars, hookah and cigarettes)	Susceptibility to four product classes (e-cigarette, cigarettes, hookah and cigars)	4 (e-cigarettes, cigarettes, hookah, cigars)	0.74	NR	Test Dimensionality (CFA)	NR	Concurrent validity (associated with e-cigarette ever use)
Browne & Todd, 2018 [38]	Fagerström Test for Nicotine Dependence applied to Vaping (FTND-V)–	E-cigarette dependence and consumption	1	.54	NR	Test Dimensionality (CFA)	NR	NR
Hershberger et al., 2017 [24]; Kale et al., 2020 [25]	Comparing E-Cigarette and Cigarettes Questionnaire (CEAC)	Comparative beliefs of e-cig use and cigarette smoking	3 (general benefits, general effects; health benefits	0.94 Subscales: .80–.88	NR	Test Dimensionality (EFA & CFA); Measurement invariance; Test differences by e-cigarette use status; Associated with impulsive personality traits	NR	Concurrent validity (associated with e-cigarette use)
Waters et al., 2017 [28]	Perceived harms and social norms in the use of electronic cigarettes	Perceived harms and social norms	1 (perceived harms), 1 (social norm)	0.87 (perceived harms) .93 (social norm)	NR	Test Dimensionality (EFA)	NR	Concurrent validity (associated with e-cigarette use)
Persoskie et al., 2017 [27]	Direct and indirect measures of perceived harm of e-cigarettes and smokeless tobacco	Perceived harm of e-cigarettes	2 (direct and indirect)	NR	NR	NR	NR	Concurrent validity (associated with e-cigarette use)
Copeland et al., 2017 [22]	Risk and Benefits of E-cigarettes (RABE)	Perceived risk and benefits of e-cigarette use (RABE)	2 (perceived risks, perceived benefits)	Subscales: .89–.92	NR	Test Dimensionality (CFA); Test differences by e-cigarette use status); Correlation with Cigarette Dependent Index	NR	NR
Morean & L’Insalata, 2017 [21]	Short Form Vaping Consequences Questionnaire (S-VCQ)	Sensory vaping expectancies	4 (Negative consequences, Positive reinforcement, Negative reinforcement, Appetite/weight control)	Subscale: .85–.94	NR	Test Dimensionality (CFA); Measurement invariance; Correlation with smoking expectancy scale	NR	Concurrent validity (associated with frequency of e-cigarette use)
Penzes et al., 2016 [31]	Motivations of intention to try EC	Motivations of intention to try e-cigarette	6 (health benefits/smoking cessation; curiosity/taste variety; perceived social norms; convenience; chemical hazards; danger of dependence)	subscale: .68–.90	NR	Test Dimensionality (EFA)	NR	Concurrent validity (subscales (curiosity/taste) associated with e-cigarette experimentation)
Hershberger et al., 2016 [29]	Nicotine and Other Substance Interaction Expectancy Questionnaire E-cig Revised version (NOSIE-ER)	Expectancies of combined e-cig and alcohol use	2 (alcohol use leads to e-cigarette use, e-cigarette use leads to alcohol use)	Subscale:.84–.88 Subscale: .85–.94	NR	Test Dimensionality (EFA & CFA)	NR	Concurrent validity (associated with alcohol use disorder)
Hendricks et al., 2015 [26]	E-cigarette-specific Brief Smoking Consequences Questionnaire-Adult (BSCQ-A)	Hospitalized smokers’ expectancies for electronic cigarettes	10 (negative affect reduction, stimulation/state enhancement, health risks, taste/sensorimotor manipulation, social facilitation, weight control, craving/addiction, negative physical feelings, boredom reduction, negative social impression)	.67–.88	NR	Test Dimensionality (CFA); Correlations with tobacco use, and e-cigarette exposure and use	NR	Concurrent validity (associated with intention to use e-cigarettes)
Chaffee et al., 2015 [23]	Conditional Risk Assessment of Adolescents’ Electronic Cigarette Perceptions	Perceived risk and benefits for use of e-cigarette	2 (perceived risks and benefits)	NR	NR	Test differences by e-cigarette use status; Correlation with Cigarette Dependent Index	NR	Concurrent validity (associated with e-cigarette use)

*Abbreviations*: *CFA* Confirmatory factor analysis, *EFA* Exploratory factor analysis, *PFA* Principal factor analysis

#### Reliability

Reliability refers to the degree to which the participants’ responses are repeatable, which is often measured by test–retest. In addition, reliability is often referred to as internal consistency, meaning the degree to which the set of items in the scale vary relative to their sum score, which is often estimated by Cronbach’s alpha [42]. Internal consistency based on Cronbach’s alpha was the only reliability test used in the identified studies. None reported an item analysis or the test and retest reliability. Among the identified studies, most instruments (86.4%, 19/22) reported internal consistencies, and two studies did not report (68.2%, 15/22) were supported with the Cronbach’s alpha ≥ .70 [19, 32], although two borderline values of .67 [26] and .68 [41] were found. Two studies reported Cronbach’s alpha <.67 [38, 40]. Most studies did report domain-specific Cronbach’s alpha scores.

#### Validity

Content validity refers to the adequacy of items or content relevant to an instrument for the construct that is measured [43]. A common method to support content validity is through computing experts’ ratings of item relevance [44]. Content validity was reported in only one instrument. This study also reported the process used for the participant interviews as part of the process of the instrument development [18, 37, 40].

Construct validity determines the extent of the construct dimensions and their underlying relationships. This is often confirmed by confirmatory factor analysis. In addition, other tests, such as convergent validity, discriminant validity, correlation analysis, group different tests, can be used. Convergent validity, which examines the same concept, is measured by different instruments but yields similar results. Discriminant validity examines if different concepts are measured by different instruments as intended. Correlation analysis examines the relationship between the newly developed instrument and new instruments, and group difference tests the differences between distinct different groups [42].

Among the included studies, construct validity was reported in 86.4% (19/22) of the all instruments included (Table 3). Most studies tested the dimensionality to support construct validity using either confirmatory factor analysis (CFA) or exploratory factor analysis (EFA). Only seven instruments were tested by both EFA and CFA or principal factor analysis (PFA) and CFA as analytic methods [18, 20, 24, 29, 34, 35]. In addition, construct validity was supported by testing the correlation with existing e-cigarette use-related measures, such as dependence measures [22, 26, 35, 37, 40]. Two studies reported testing the convergence and divergence validity [27, 35]. A number of studies reported measurement invariance in testing the instrument [21, 34], as well as construct validity in order to test differences in e-cigarette use status [22, 23].

Criterion validity is to ensure the instrument measures the latent dimension as intended, which is often tested with predictive validity and concurrent validity. Those tests determine whether the score of the instrument predicts or has a strong relationship with the outcome measures or criterion measures. In this case, studies that tested whether the construct of the instrument predicted future e-cigarette use behaviors (predictive validity) or have a significant association between e-cigarette use and the construct of the instrument (concurrent validity) were considered that they checked criterion validity. Criterion validity was reported by 77.3% (17/22) of the included articles (Table 3). Most studies reported either the concurrent validity or predictive validity. For concurrent validity, the associations with existing measures such as e-cigarette use or e-cigarette experimentation were tested and reported acceptable criterion validity [18, 20, 23, 24, 26–28, 41]. For predictive validity, four instruments were tested in two studies to explore whether the constructs of the instrument measures would predict positive future e-cigarette use [32, 40].

### Quality appraisal

The overall quality of the studies based on the Cosmin Risk of Bias checklist varied (Table 4). Most studies had problems with the PROM design criteria. Studies should provide clearer description of the constructs to be measured with the theoretical framework. In addition, most studies did not test for content validity through qualitative methods. Structural validity was supported by CFA and EFA. However, a few studies only conducted EFA, but needed to conduct CFA as well. For internal consistency, most studies reported Cronbach’s alpha based on subscales, but a few studies only reported Cronbach’s alpha for the whole scale, even when they were not measuring unidimensional construct. In addition, most studies did not report reliability with appropriate methods, such as the intraclass correlation coefficient or Kappa score. In addition, most studies did not check measurement error, and responsiveness, and studies need to check criterion validity and measurement invariance.

**Table 4:** Quality of appraisal and risk of bias

	PROM design	Content validity	Structural validity	Internal consistency	Cross-cultural validity/ Measurement invariance	Reliability	Measurement error	Criterion Validity	Hypotheses Testing for Construct Validity	Responsiveness
Cristello et al., 2020 [19]	Inadequate	Inadequate	Very good	Inadequate	Inadequate	Inadequate	Inadequate	Inadequate	Very good	Inadequate
Diez et al., 2019 [30]	Inadequate	Inadequate	Very good	Very good	Inadequate	Inadequate	Inadequate	Inadequate	Very good	Inadequate
Morean et al., 2019 [20]	Inadequate	Inadequate	Very good	Very good	Very good	Inadequate	Inadequate	Very good	Very good	Inadequate
Piper et al., 2019 [36] (e-FTCD)	Inadequate	Inadequate	Very good	Very good	Inadequate	Inadequate	Inadequate	Very good	Very good	Inadequate
Piper et al., 2019 [36] (e-WISDM)	Inadequate	Inadequate	Very good	Very good	Inadequate	Inadequate	Inadequate	Very good	Very good	Inadequate
Piper et al., 2019 [36] (PS-ECDI)	Inadequate	Inadequate	Very good	Very good	Inadequate	Inadequate	Inadequate	Very good	Very good	Inadequate
Foulds et al., 2015 [37]	Inadequate	Very good	Inadequate	Inadequate	Inadequate	Inadequate	Inadequate	Very good	Very good	Inadequate
Dowd et al., 2019 [35]	Inadequate	Inadequate	Very good	Very good	Inadequate	Inadequate	Inadequate	Inadequate	Very good	Inadequate
Dowd et al., 2019(short) [35]	Inadequate	Inadequate	Very good	Very good	Inadequate	Inadequate	Inadequate	Inadequate	Very good	Inadequate
Cole et al., 2019 [32]	Inadequate	Inadequate	Inadequate	Inadequate	Inadequate	Inadequate	Inadequate	Very good	Inadequate	Inadequate
Pokhrel et al., 2018 [18]	Adequate	Inadequate	Very good	Very good	Inadequate	Inadequate	Inadequate	Very good	Very good	Inadequate
Pokhrel et al., 2018(short) [18]	Adequate	Inadequate	Very good	Very good	Inadequate	Inadequate	Inadequate	Very good	Very good	Inadequate
Morean et al., 2018 [34]	Inadequate	Inadequate	Very good	Very good	Very good	Inadequate	Inadequate	Very good	Very good	Inadequate
Carey et al., 2018 [33]	Inadequate	Inadequate	Very good	Inadequate	Inadequate	Inadequate	Inadequate	Very good	Very good	Inadequate
Browne & Todd, 2018 [38]	Inadequate	Inadequate	Very good	Very good	Inadequate	Inadequate	Inadequate	Inadequate	Very good	Inadequate
Hershberger et al., 2017 [24]; Kale et al., 2020 [25]	Adequate	Inadequate	Very good	Very good	Very good	Inadequate	Inadequate	Very good	Very good	Inadequate
Waters et al., 2017 [28]	Adequate	Inadequate	Adequate	Very good	Inadequate	Inadequate	Inadequate	Very good	Inadequate	Inadequate
Persoskie et al., 2017 [27]	Inadequate	Inadequate	Inadequate	Inadequate	Inadequate	Inadequate	Inadequate	Very good	Inadequate	Inadequate
Copeland et al., 2017 [22]	Inadequate	Inadequate	Doubtful	Very good	Inadequate	Inadequate	Inadequate	Inadequate	Very good	Inadequate
Morean & L’Insalata, 2017 [21]	Adequate	Inadequate	Very good	Very good	Inadequate	Inadequate	Inadequate	Very good	Very good	Inadequate
Penzes et al., 2016 [31]	Inadequate	Inadequate	Inadequate	Inadequate	Inadequate	Inadequate	Inadequate	Very good	Inadequate	Inadequate
Hershberger et al.,2016 [29]	Inadequate	Inadequate	Very good	Very good	Inadequate	Inadequate	Inadequate	Very good	Very good	Inadequate
Hendricks et al., 2015 [26]	Adequate	Very good	Inadequate	Inadequate	Inadequate	Inadequate	Inadequate	Very good	Very good	Inadequate
Chaffee et al., 2015 [23]	Adequate	Inadequate	Inadequate	Inadequate	Inadequate	Inadequate	Inadequate	Very good	Very good	Inadequate

## Discussion

This paper is the first systematic review of existing instruments on e-cigarette related constructs. A total of 23 studies were identified that focus on the development or validation of 22 instruments. This study provides an overview of these instruments as well as development process, theoretical framework, target population, and psychometric properties. This review can serve as a useful guide for healthcare professionals and researchers seeking to conduct assessments or conduct research into the phenomenon of e-cigarette use.

In this review, we identified several e-cigarette related constructs in existing instruments. Beliefs or perceptions about e-cigarettes were considered the most commonly studied determinants of current e-cigarette use based on the validated instruments. To explore beliefs or perceptions, constructs including outcome expectancies, sensory expectancies, and perceived risks and benefits were explored. These constructs were supported by motivation theories, social learning theory, and the theory of planned behavior. In addition, beliefs or perceptions about the relative merits of e-cigarettes and smoking conventional cigarettes were another commonly explored construct. In terms of the motivations for e-cigarette experimentation and susceptibility to future use were constructs explored, although habitual e-cigarette use was the only construct used to assess current e-cigarette use. The consequences or symptoms related to e-cigarette use were also explored with the constructs of e-cigarette craving and dependence. Only a few studies included in this review provided a theoretical background of the instrumentation and did not clearly present the conceptual framework or definition. This may be related to potential issues of clarity of the constructs that each instrument measures. The constructs that each instrument intends to measure may not be specific enough without a theoretical guide [45]. In addition, most studies only described the validation process but did not provide detailed steps of the development process of the instruments, which also limits to clarify constructs that each instrument intended to measure. Interestingly, there were two instruments available for the outcome expectancies although the target populations were different. Three different instruments existed for e-cigarette dependence; however, only one instrument was supported by content, construct, and criterion validity. This may indicate that there is not yet a consensus, and further studies are needed to test the validity of these instruments through comparison studies to draw results that are more accurate.

Regarding reliability, most studies reported acceptable internal consistencies. However, reliability was supported by only one type of reliability and tested the internal consistencies with a single method. This can be a potential threat of internal consistency [46]. It is suggested that three broad types of reliability need to be assured, including (1) reliability from administering parallel forms of instruments (alternate-form coefficients), (2) reliability from administering the same instrument on separate times (test-retest), and (3) reliability based on total scores or subsets of items within a single test (internal consistency coefficient) [47]. It is important to test multiple types of reliability by multiple methods, such as “test-retest” or “item-analysis”. In this way, any systematic error or variations of instruments can be prevented and the generalizability of the use of instruments can be improved.

For validity, only a limited number of studies conducted both EFA and CFA during their analysis, which again limits the construct validity, and few tested either content validity or criterion validity. Part of the reason why content validity was not tested in most studies may be related to the historical aspects of the development process commonly used for e-cigarette-related measures, most of which are based on existing instruments originally developed to assess cigarette smoking related constructs. As these measures have already been extensively validated, the various authors have simply adapted these for e-cigarette specific constructs, not considering this to be a necessary step in the development process. However, it is actually important to capture the unique aspects of e-cigarettes, which are in many ways very different from cigarettes [45]. Moreover, in terms of the criterion validity, although most studies did provide a test of the criterion validity, only a limited number also tested the predictive validity. This seriously limits the validity of the majority of the existing e-cigarette related instruments [42].

### Limitations

As always with studies of this nature, there is a risk that relevant articles may have been missed even though we have used a range of different techniques to systematically search for articles; there is also the potential for errors to occur in the review and coding process. To minimize these errors and ensure the reliability of the coding process, two researchers independently coded the articles, and three researchers double checked the accuracy of the coding multiple times. Where discrepancies were identified, three authors reviewed the articles together and came to a consensus. Further review from other researchers would have been considered to deal with any unresolved issues had any such occurred, but this was not found to be necessary.

Moreover, there is also a possibility that not all studies were able to report the full details of their instrumentation or validation process due to limited space in peer-reviewed journals. It is thus possible that the authors were not able to gather sufficient information for each measure in this review from the published reports.

### Recommendations for practice

Twenty-two unique instruments assessing the constructs related to e-cigarette use in population studies were identified in this study. Our findings suggest that practitioners should first consider choosing instruments based on the constructs that they are most interested in, depending on the purpose of the assessment. For example, if practitioners are interested in the reasons for e-cigarette use, it would be most appropriate to select an instrument that assesses various types of beliefs or perceptions, while to assess non-users’ motivation or susceptibility to future use, they can choose from three different measures: motivations for e-cigarette experimentation [41], a susceptibility scale [32], and susceptibility to four product classes (e-cigarettes, cigars, hookah and cigarettes) [33]. If they are interested in current habitual use, one instrument has been specifically developed to study this, the Self-Report Habit Index (SRHI) [21], and if the practitioners need to assess the symptoms of current e-cigarette users, a number of measures are available to assess craving or dependence, namely the Questionnaire of Vaping Craving (QVC) [35], the Penn State Electronic Cigarette Dependence Index (PS-ECDI) [37, 40], the E-cigarette Fagerström Test of Cigarette Dependence (e-FTCD) [11], the E-cigarette Wisconsin Inventory of Smoking Dependence Motives (e-WISDM) [11], and the Fagerström Test for Nicotine Dependence applied to Vaping (FTND-V) [38].

After narrowing down the broad categories of constructs depending on the purpose of the assessment, practitioners should consider the age of their target population and make sure that the instrument has been validated for this user group. Among those instruments, those that report a value of Cronbach’s alpha higher than .70 and support both construct and criterion validity should be preferred, although the number of items should be considered to determine the feasibility of their use in clinical settings.

### Recommendations for future research

Future work on survey instruments measuring e-cigarette-related constructs that take into account content validity and criterion validity will be necessary if we are to establish a stronger evidence base for e-cigarette research. Currently, most studies report only construct validity, with few also reporting the content validity or predictive validity. It is important that multiple types of reliability in addition to Cronbach’s alpha alone need to be explored and supported when a new instrument is developed. In addition, for the instruments reported low reliability coefficients, item modification is needed to ensure a desirable internal consistency [45, 47].

There is a critical need to develop a reliable and valid instrument with which to assess e-cigarette-related constructs of diverse populations. Currently, only a limited number of instruments assessing e-cigarette related constructs have been validated for adolescent populations that have been specifically designed to assess the perceived risks and benefits of e-cigarettes, their perceived harms compared with cigarettes, and susceptibility to future use. However, among the available instruments for this age group, only one study was reported as having an acceptable internal consistency. Given the dramatic increase in the prevalence of e-cigarette use, there is clearly a need to develop and validate instruments targeted specifically at adolescents and young adults, particularly given that these are the people most likely to be using e-cigarettes. The availability of such an instrument will enhance the rigor of research on e-cigarettes and help us understand the rapid growth in the popularity of e-cigarettes among this population. Furthermore, instruments need to be validated in diverse clinical settings, and there is also a need for validated universal instruments that can be administered across age groups to help us understand the impact of differences in the various associated factors, the characteristics of the different types of e-cigarettes, and the symptoms across both clinical and non-clinical groups.

Providing details of the instrument administration is also important for researchers and practitioners. Currently, only a few articles provide basic information on the characteristics of their instruments, such as the mode and response type. It is important for researchers to report detailed information, such as whether the instrument is provided online or on paper, the various response options, and the number of items to make the instruments more suitable for use by practitioners and other researchers alike.

## Conclusions

This systematic review provides a critical appraisal and repository of the instruments measuring e-cigarette-related constructs in the current literature. It serves as a user-friendly guide to help researchers select the most appropriate instrument to suit their needs based on the constructs, target population, psychometric properties, and number of items, all of which can help develop a more accurate understanding of e-cigarette related phenomena for practitioners. For future studies, researchers need to expand the validation of the existing instruments to include more diverse populations, and develop new instruments that are specific to the unique aspects of e-cigarettes. The development of instruments capable of assessing different aspects of e-cigarette use with a strong theoretical background and validation process will be essential to support efforts to develop effective e-cigarette use prevention and cessation programs.

## Data Availability

The data that support the fin available used and/or analyzed during the current study are available from the corresponding author on reasonable request.

## Abbreviations

E-cigarettes: Electronic cigarettes
CINAHL: Cumulative Index of Nursing and Allied Health Literature
EMBASE: Excerpta Medica dataBASE
ENDS: electronic nicotine delivery systems
PRISAMA: Preferred Reporting Items for Systematic Reviews and Meta-Analyses
MeSH: Medical Subject Headings
CFA: Confirmatory Factor Analysis
EFA: Exploratory factor analysis
PFA: principal factor analysis
SRHI: Self-Report Habit Index
QVC: Questionnaire of Vaping Craving
e-FTCD: E-cigarette Fagerström Test of Cigarette Dependence
e-WISDM: E-cigarette Wisconsin Inventory of Smoking Dependence Motives
FTND-V: Fagerström Test for Nicotine Dependence applied to Vaping
